# Unraveling the role of PBK in glioblastoma: from molecular mechanisms to therapeutic targets

**DOI:** 10.1097/MS9.0000000000002708

**Published:** 2024-11-04

**Authors:** Yizheng Zhang, Mingyuan Luan

**Affiliations:** Medicine Faculty, University of Tübingen, Tübingen, Germany

**Keywords:** bioinformatics, eukaryotic initiation factor 4E, glioblastoma multiforme, glioma initiating cell, glioma, PDZ-binding kinase

## Abstract

**Background:**

This study investigates the gene expression characteristics of glioma-initiating cells (GIC), an important subgroup of glioblastoma (GBM), after knockdown of PBK (PDZ-binding kinase). Differentially expressed genes (DEGs) between PBK knockdown GIC and control groups were screened through bioinformatics methods. The authors analyzed the mechanisms and roles of these DEGs in GBM tumorigenesis and patient prognosis.

**Methods:**

Microarray data (GSE53800) were obtained from the Gene Expression Omnibus (GEO) database, selecting 18 GIC cell line samples with or without PBK knockdown. Each control and knockdown group contained three samples. DEGs were screened using R software. GO enrichment analysis, KEGG pathway analysis, PPI network analysis, and hub gene identification were conducted to explore DEG mechanisms. Western blot analysis was also performed to detect EIF4E protein expression, one of the key hub genes, after PBK knockdown in the HS683 glioma cell line.

**Results:**

A total of 175 upregulated and 145 downregulated genes were identified. GO analysis showed that DEGs were mainly enriched in the positive regulation of cell proliferation, cell adhesion, and angiogenesis. KEGG pathway analysis revealed that DEGs were mainly involved in neuroactive ligand-receptor interactions, calcium signaling, and HIF-1 signaling pathways. Western blot results indicated that EIF4E was downregulated after PBK knockdown.

**Conclusion:**

A group of genes, such as EIF4E, were closely associated with PBK expression and functions. These findings may provide insight into the molecular mechanism of PBK in GBM.

## Introduction

Highlights

- Investigated the role of PDZ-binding kinase (PBK) in glioblastoma multiforme (GBM) and its potential as a therapeutic target.
- Identified 320 differentially expressed genes (DEGs) in PBK knockdown GICs, affecting cell proliferation, adhesion, and angiogenesis.
- Highlighted key signaling pathways, including neuroactive ligand-receptor interaction, calcium signaling, and HIF-1, while showing that PBK knockdown significantly reduced eukaryotic initiation factor 4E (EIF4E) expression, a crucial gene in tumorigenesis.
- Provided new insights into PBK’s molecular mechanisms and potential therapeutic targets for GBM.

GBM is the most common and fatal nervous system tumor in adults^1^. Its incidence is 3.19 per 100 000^2^, and GBM patient prognosis is poor, with only a few surviving beyond 2.5 years after diagnosis, and a 5-year survival rate of less than 5%^3^. Surgical resection combined with postoperative chemotherapy is the recommended treatment^4,5^, but survival rates have not improved significantly in the past 30 years^3,6^. Therefore, exploring new GBM treatments is crucial. GIC is a special GBM subgroup with some characteristics of normal neural stem cells^7^. GIC is relatively insensitive to chemotherapy and radiotherapy and plays a role in cell growth, angiogenesis, and invasion^8,9^. Consequently, our research aims to identify molecular mechanisms and signaling pathways in GIC to offer insights into potential therapeutic targets.

PBK, a protein-threonine kinase, belongs to the MAPKK (mitogen-activated protein kinase kinase) family and regulates the cell cycle and mitosis^10,11^. PBK is highly expressed in GIC and GBM^7,12^, with significantly higher expression in high-grade gliomas (grade III or IV) compared to low-grade gliomas (grade II)^13^. Elevated PBK expression may be linked to greater tumor malignancy and worse prognosis^14,15^. Studies show that a positive feedback loop between PBK and ERK leads to uncontrolled tumor cell proliferation^16^. In some cases, aberrant PBK expression promotes tumor cell migration via the PI3K/PTEN/AKT pathway^15^, enhancing invasiveness by stabilizing the oncoprotein c-MYC^17^. PBK is also linked to autophagy and chemotherapy resistance^18^. PBK knockdown increases GBM sensitivity to temozolomide^13^, indicating that PBK activation contributes to chemotherapy resistance, weakening treatment efficacy and reducing survival. Therefore, studying PBK-related molecules and pathways is vital for identifying potential GBM treatment targets.

The Gene Expression Omnibus (GEO) enables researchers to upload and download high-throughput gene expression profiles and other genomic data^19^. We performed bioinformatics analysis on microarray datasets from GEO. By selecting and grouping microarray research samples, we identified DEGs and analyzed their mechanisms, shedding light on potential therapeutic targets and providing clinical treatment insights.

## Materials and methods

### Data processing and differential gene screening

We downloaded the gene expression profile dataset GSE53800 from the GEO database (http://www.ncbi.nlm.nih.gov/geo/), based on the GPL10558 platform (Illumina HumanHT-12 V4.0 expression beadchip, Illumina). A total of 18 samples, with or without PBK knockdown, from two GIC cell lines (T08 and T65) were selected. Each control and knockdown group contained three samples. Background correction, homogenization, and DEG screening were conducted using the limma package in R software (3.5.0, https://www.r-project.org). Statistical data were analyzed using *t*-tests and Bayesian tests, with a |log2FC| > 1 and adjusted *P*-value <0.05 as the screening threshold. A Venn diagram was drawn using the Venn Diagram package (http://bioinformatics.psb.ugent.be/webtools/Venn/).

### GO and KEGG enrichment analysis

We used DAVID (http://david.abcc.ncifcrf.gov/) for gene ontology (GO) and Kyoto Encyclopedia of Genes and Genomes (KEGG) enrichment analysis, using *P*<0.05 as the selection criterion. GO and KEGG were visualized using the ggplot2 package.

### Construction of the protein–protein interaction network

The STRING database (https://string-db.org) analyzed and predicted protein interactions. DEGs were imported into STRING to generate the PPI network structure diagram, and Cytoscape 3.6.1 (http://www.cytoscape.org) was used for editing and visualization. The MCODE plugin clustered the PPI network based on topology to identify the most significant module.

### Hub genes identification and analysis

The Maximal Clique Centrality (MCC) algorithm, via the cytoHubba plugin in Cytoscape, identified hub genes. The online database cBioPortal (http://www.cbioportal.org) analyzed co-expression relationships between genes^22^. GEPIA (gepia.cancer-pku.cn) analyzed gene expression differences between GBM and normal tissues and performed survival analysis^23^.

### Cell culture

The human glioma cell line HS683 was obtained from the American Type Culture Collection (ATCC) and cultured according to ATCC instructions in DMEM medium with 10% FBS in a 5% CO_2_ incubator at 37°C. shRNA sequences for PBK knockdown and cell line establishment were based on a previous study^13^.

### Western blot

We inoculated 2×10^6 cells into each 10 cm dish. After cell confluence reached 70–80%, cells were lysed using 300 μl RIPA buffer. Protein samples (50–100 μg) were separated using 10% SDS-PAGE and transferred to PVDF membranes. Primary and secondary antibodies were incubated sequentially, and chemiluminescence was used for development. Anti-PBK and anti-β-actin were purchased from Santa Cruz (Santa Cruz), and anti-eIF4E was purchased from Proteintech (Wuhan, Hubei, China).

### Ethical considerations

This study used publicly available databases with decoded and de-identified data, ensuring no involvement of patient or animal subjects. Therefore, ethical approval from an institutional review board was not necessary.

## Results

### Identification of DEGs between PBK knockdown and PBK NS glioma-initiating cells

**Figure 1:**
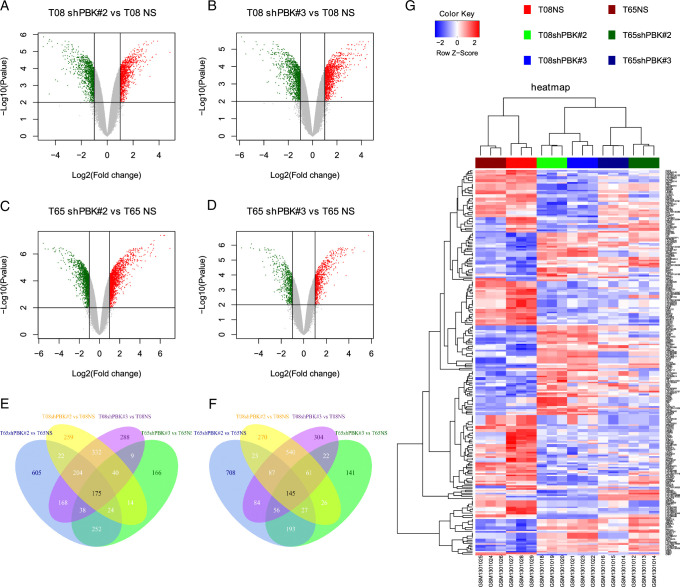
Identification of DEGs between PBK knockdown and PBK NS glioma initiating cell. DEGs were selected with |log_2_FC| > 1 and adj. *P*-value <0.05 in the mRNA expression profiling dataset GSE53800 (A-D). Venn diagrams showed overlaps of 175 upregulated genes (E) and 145 downregulated genes (F) among four independent groups. Hierarchical clustering heatmap was constructed. Upregulated genes are marked in red and downregulated genes are marked in blue (G).

In this study, raw data from PBK knockdown and control GIC samples were extracted from the GSE53800 dataset in the NCBI-GEO database. The DEG screening process is shown in volcano plots (Fig. 1A–D) under the screening criteria of |log2FC| > 1 and adjusted *P*-value <0.05. We identified 175 upregulated genes (Fig. 1E) and 145 downregulated genes (Fig. 1F). A hierarchical clustering heatmap provided an overview of DEG expression (Fig. 1G).

### GO and KEGG pathway enrichment analysis of DEGs

**Figure 2:**
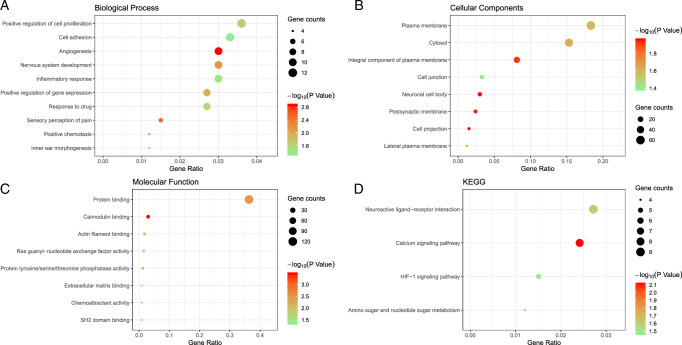
GO and KEGG pathway enrichment analysis of DEGs. Biological processes (A). Cellular components (B). Molecular function (C). KEGG pathway (D). The sizes and color of bubbles indicate the number of involved genes and their significant level.

GO analysis showed DEGs were enriched in positive regulation of cell proliferation, cell adhesion, and angiogenesis (Fig. 2A). KEGG pathway analysis revealed neuroactive ligand-receptor interactions, calcium signaling, and HIF-1 signaling pathways were significantly enriched (Fig. 2D).

### PPI network construction and hub gene identification

**Figure 3:**
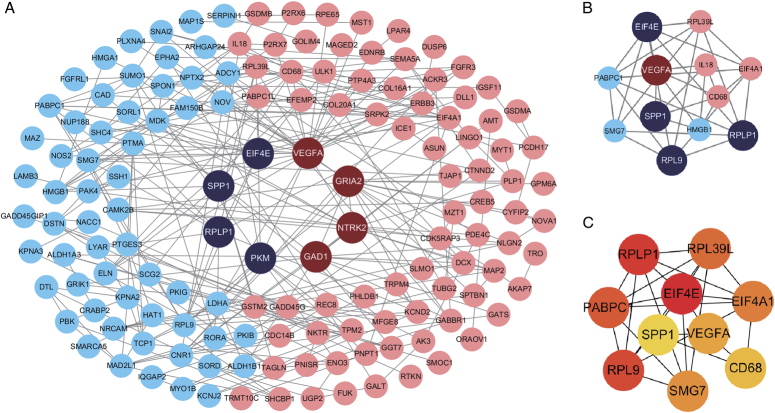
Construction of protein–protein interaction network and identification of hub genes. One hundred forty-five genes were finally enrolled and PPI network was constructed by Cytoscape. Upregulated genes were marked in red and downregulated genes were marked in blue. Nodes with degree >10 were highlighted in deep red or deep blue (A). The most significant module was obtained from the PPI network with 12 nodes and 32 edges (B). The top 10 hub genes were identified by MCC method (C).

The PPI network, generated by STRING and Cytoscape, marked upregulated genes in red and downregulated ones in blue. Nodes with degrees >10 were highlighted (Fig. 3A). The MCODE plugin generated the most significant module, containing 12 nodes and 32 edges (Fig. 3B). Functional enrichment analysis revealed genes in the most significant module were enriched in transcription initiation, ribosomal large subunits, and RNA transport (Table 1). CytoHubba’s MCC method identified the 10 most significant hub genes (Fig. 3C), all related to neoplasm in various ways (Table 2).

**Table 1:** Functional roles of top 10 hub genes.

Gene symbol	MCC score	Full name	Function
EIF4E	257	Eukaryotic translation initiation factor 4E	EIF4E promotes tumorigenesis via translational control^41^
RPLP1	247	60S acidic ribosomal protein P1	RPLP1 promotes tumor metastasis of triple-negative breast cancer^44^
RPL9	245	L ribosomal proteins	the growth of CRC can be suppressed by knockdown of RPL9^45^
PABPC1	244	Polyadenylate-binding protein 1	reduced expression of PABPC1 was associated with tumor progression and poor prognosis in esophageal cancer^46^
RPL39L	242	Ribosomal protein L39 like	the expression of Rpl39l strongly correlates with high tumor grading of HCC samples and AFP level^47^
EIF4A1	125	Eukaryotic initiation factor 4A-I	Silencing eIF4A1 decreases proliferation and invasion of melanoma^48^
SMG7	124	Protein SMG7	SMG7 takes part in p53-mediated p21 activation and cell cycle arrest^49^
VEGFA	78	Vascular endothelial growth factor A	VEGFA mediates angiogenesis and tumorigenesis of many types of cancer^50^
CD68	39	Macrosialin	densities of CD68 positively correlated with postoperational overall survival of esophageal squamous cell carcinoma patients^51^
SPP1	37	Osteopontin	SPP1 sustains glioma cell survival and stimulates angiogenesis^52^

AFP, alpha-fetoprotein; CRC, colorectal cancer; HCC, hepatocellular carcinoma.

**Table 2:** GO and KEGG pathway enrichment analysis of DEGs in the most significant module.

Category	ID	Description	Count	*P*
GOTERM BP	GO:0006413	translational initiation	5	1.34E-06
GOTERM BP	GO:0000184	nuclear-transcribed mRNA catabolic process, nonsense-mediated decay	4	5.49E-05
GOTERM BP	GO:0000289	nuclear-transcribed mRNA poly(A) tail shortening	3	1.57E-04
GOTERM BP	GO:0032147	activation of protein kinase activity	3	3.80E-04
GOTERM CC	GO:0022625	cytosolic large ribosomal subunit	3	7.38E-04
GOTERM CC	GO:0016281	eukaryotic translation initiation factor 4F complex	2	0.005420486
GOTERM CC	GO:0005829	cytosol	7	0.007206609
GOTERM MF	GO:0005125	cytokine activity	4	1.27E-04
GOTERM MF	GO:0005515	protein binding	11	0.001453344
GOTERM MF	GO:0000339	RNA cap binding	2	0.005909625
GOTERM MF	GO:0003735	structural constituent of ribosome	3	0.007227332
KEGG PATHWAY	hsa03013	RNA transport	3	0.024514491
KEGG PATHWAY	hsa04151	PI3K-Akt signaling pathway	3	0.08647498

BP, biological processes; CC, cellular components; GO, gene ontology; KEGG, Kyoto Encyclopedia of Genes and Genomes; MF, molecular function.

### EIF4E upregulation, co-expressed with PBK, affects patient survival

**Figure 4:**
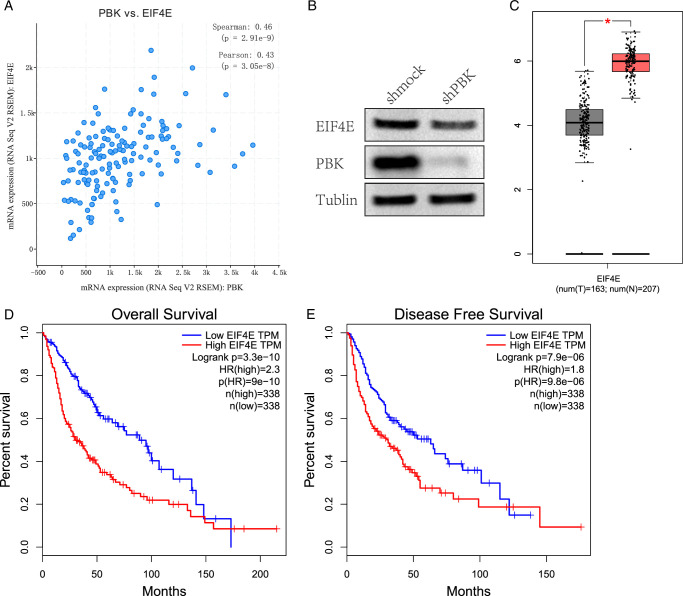
Association between EIF4E and PBK and patient survival. The co-expression relationship between EIF4E and PBK was performed by the cBioPortal online platform (A). The expression of EIF4E was downregulated after PBK knockdown in HS683 (B). GEPIA online platform was used to perform the expression of EIF4E in glioma and its effect on survival. EIF4E mRNA expression in GBM compared to normal brain tissues (C) in which normal samples are marked in gray and GBM samples are marked in red. High EIF4E expression decreases both overall survival (D) and disease-free survival (E).

EIF4E scored highest in the MCC algorithm and had a high degree value. A co-expression relationship between EIF4E and PBK in GBM was revealed through the cBioPortal database (Fig. 4A). Western blot results showed a significant reduction in EIF4E expression after PBK knockdown in glioma cell line HS683 (Fig. 4B). The GEPIA database indicated EIF4E expression was significantly higher in GBM than in normal tissue (Fig. 4C). Survival analysis showed shorter overall survival and disease-free survival in glioma patients with high EIF4E expression (Fig. 4D, E).

## Discussion

PBK is a protein serine/threonine kinase that contains a homologous sequence of the MAPKK (mitogen-activated protein kinase kinase) family and is functionally similar to MEK3/6, which activates the p38-MAPK pathway^24^. Aberrant PBK expression occurs in various tumors and is closely related to tumor cell proliferation, metastasis, tumor grading, and patient prognosis^12,18,25,26^. A positive feedback loop between PBK and ERK was identified in the colorectal cancer cell line HCT116, resulting in uncontrolled cell proliferation. Meanwhile, this activation was not inhibited by the negative feedback effects caused by b-raf and c-raf^16^. In breast cancer cell lines, the CDK/cyclin B1 complex can increase the phosphorylation level of PBK by inhibiting protein phosphatase alpha-1, thereby promoting mitosis^27^. In the central nervous system, PBK-positive cells are found in rapidly proliferating early postnatal cerebellar granules, the adult subventricular zone, and glial fibrillary acidic protein (GFAP)-positive neural stem cells, while PBK expression is significantly reduced in mature neurons^11^. More importantly, PBK expression is much higher in GBM than in normal tissue and is significantly correlated with glioma grade. Additionally, a study that isolated GICs from glioma for primary culture found that PBK mRNA and protein levels were significantly upregulated^7^. In summary, PBK promotes tumor proliferation and metastasis and is closely related to tumor grading and patient prognosis. It has also been confirmed as an important biomarker of GBM. However, the molecular mechanism of PBK in GBM has not yet been fully studied. It remains unclear what biological functions and signaling pathways PBK may be involved in during GBM development. In fact, the survival rate of GBM patients has not significantly improved in the past 30 years. To address this, expanding the limited treatment options is particularly important. The development of new treatments depends on a deeper understanding of tumor molecular mechanisms. Therefore, PBK, as a biomarker of GBM, holds significant importance and broad potential for research into its molecular mechanisms.

In this study, we performed a grouped analysis of mRNA microarray datasets to analyze DEGs between GICs with or without PBK knockdown. A total of 320 DEGs were identified, including 175 upregulated genes and 145 downregulated genes. To explore the correlation between DEGs and their primary functions, we conducted GO and KEGG enrichment analysis. GO enrichment analysis revealed that DEGs are mainly enriched in positive regulation of cell proliferation, cell adhesion, and angiogenesis. The major changes in KEGG pathway enrichment were neuroactive ligand-receptor interactions, calcium signaling, and HIF-1 signaling pathways. Among these, angiogenesis has been shown to be involved in the development and progression of various tumors^28–30^. HIF-1 is a transcriptionally active protein regulated by hypoxia signaling, involved in tumorigenesis and proliferation^31,32^, and also promotes angiogenesis^33^. Additionally, the positive regulation of cell proliferation plays a significant role in tumor cell proliferation, while cell adhesion is critical for tumor cell invasion and migration. Furthermore, it has been reported that disruptions in calcium signaling can lead to uncontrolled tumor cell proliferation and resistance to cell death^34^. In short, the literature suggests that PBK is a gene closely related to tumor development, and altering its expression will inevitably lead to changes in tumor-related biological phenotypes and molecular signaling pathways, consistent with our data and analysis.

We identified the top 10 hub genes, among which EIF4E received the highest score using the MCC method. EIF4E interacts directly with other hub genes: VEGFA, PABPC1, SMG7, RPL9, RPLP1, EIF4A1, and RPL39L in the most significant module of the PPI network. GO and KEGG enrichment analysis of the most significant modules indicated that these genes, including EIF4E, are mainly involved in translation initiation, RNA transport, and the PI3K-AKT signaling pathway. EIF4E is upregulated in GBM and other tumors^35,36^, and it promotes the translation of tumor-associated mRNAs such as VEGFA, c-Myc, and cyclin D1^37^, resulting in tumor resistance, recurrence, and shorter survival^38–40^. The activation of EIF4E is primarily mediated by the PI3K/AKT/mTOR and Ras/MAPK/Mnk signaling pathways^41^. Ras, ERK, AKT, and other key kinases in these pathways are closely related to PBK^16,42,43^. Based on this, we hypothesized that PBK may regulate the expression and activity of EIF4E, suggesting that the PBK/EIF4E pathway is likely to be an important tumor-associated pathway. While both PBK and EIF4E have been confirmed to be closely related to tumors, no studies have yet reported any direct or indirect regulatory relationship between them. In our study, GEPIA analysis revealed that EIF4E expression is significantly increased in GBM, and survival analysis showed that glioma patients with high EIF4E expression had shorter overall survival and disease-free survival, indicating that EIF4E, like PBK, could be regarded as a biomarker for glioma. Moreover, literature searches revealed that the other hub genes are also associated with tumor development^41,44–52^, though the relationship between PBK and these hub genes has not yet been further studied. Thus, exploring the relationship between PBK and hub genes like EIF4E would be of great significance.

## Conclusion

In conclusion, this study was designed to explore the DEGs potentially regulated by PBK, analyze the effects of these DEGs on tumor formation and progression, and evaluate their value as biomarkers. A total of 175 upregulated genes, 145 downregulated genes, and 10 hub genes were identified, which may function downstream of the PBK signaling pathway in GBM. However, whether PBK directly interacts with these DEGs and whether similar relationships exist between PBK and these DEGs in other tumors require further investigation.

## Ethical approval

This study did not involve the need for ethical approval.

## Consent

Informed consent signed by the patients was not required in this study.

## Source of funding

None.

## Author contribution

Y.Z.: designed this study and collected the data; Y.Z. and M.L.: analyzed the data and wrote the paper.

## Conflicts of interest disclosure

The authors declare no conflicts of interest.

## Research registration unique identifying number (UIN)

This study does not involve research on patients.

## Guarantor

Yizheng Zhang and Mingyuan Luan.

## Data availability statement

The authors are willing to provide raw data if needed.
